# Continued changes to contact lens practice following adjustments made as a result of the COVID-19 pandemic

**DOI:** 10.1016/j.heliyon.2024.e41376

**Published:** 2024-12-19

**Authors:** Federica Scarci, Alfredo Desiato, Giulia Carlotta Rizzo, Stefano Livi, Mara Marini, Alessandra Cecalupo, Silvia Tavazzi, Shehzad A. Naroo, Fabrizio Zeri

**Affiliations:** a Department of Education, Roma Tre University, Rome, Italy; b Optometry & Vision Science Research Group (OVSRG), School of Optometry, Aston University, Birmingham, UK; c University of Milano-Bicocca, Department of Materials Science, Milan, Italy; d University of Milano-Bicocca, COMiB Research Centre in Optics and Optometry, Milan, Italy; e Department of Social and Developmental Psychology, University of Rome Sapienza, Rome, Italy; f Department of Neuroscience, Imaging and Clinical Sciences, "Gabriele D'Annunzio" University of Chieti-Pescara, Chieti, Italy

**Keywords:** Contact lenses, COVID-19, Health belief model, Planned behaviour

## Abstract

**Background:**

This study aimed to explore behaviour of practitioners in response to the COVID-19 pandemic, analysing the factors that influenced their decisions to resume professional practice post-lockdown and intention to adhere to COVID-19 protective measures.

**Methods:**

A web-based cross-sectional survey targeting Italian practitioners was carried out to study the post lockdown behaviour and future intention to provide new contact lens (CL) fittings, and the use of protective measures. The survey also explored the dimensions of the Integrated Behavioural Model (IBM) and the Health Belief Model (HBM) to predict the intention to resume professional practice and to comply with protective measures respectively.

**Results:**

A total of 212 professional (150 males; age range 22–76 years) completed the survey. Past behaviour and positive attitudes toward CL practice were the variable in IBM that predicted more strongly the intention to provide both new CL fittings and aftercare visits, whereas past behaviour and perceived benefits of HBM were the strongest predictors of future compliance with protective measures.

**Conclusion:**

Results suggest that the more engaged practitioners are more willing to get back to their routine even in uncertain circumstances, and that a tailored approach, leveraging past behaviours and perceived benefits, could enhance compliance strategies.

## 1 Introduction

In the field of eyecare, there is an increasing focus on developing current, evidence-based clinical parameters that enable eye care practitioners (ECPs) to offer patients, including those who wear contact lenses (CLs), the best possible care whilst maintaining safety and effectiveness of the treatment administered [[1], [2], [3]]. However, the public does not always benefit directly and immediately from the availability of improved protocols, as the implementation of new procedures may be restricted by practitioners' reluctance for revised procedures, suggesting the need for specific training appearing to be crucial in adopting the changes [4]. The need of training has been advocated by the practitioners, who demonstrated the willing to improve their CL practice by updating their knowledge and skills, and to able to better manage the complications associated with CL wear [5]. Nonetheless, the development of clinical guidelines should consider also factors other than stakeholders’ knowledge/expertise, and include prior experience, beliefs and perceived impact of the interventions [6].

The COVID-19 pandemic deeply stressed the importance to adopt behaviours aiming to reduce potential risks to patients' and professionals’ health, while guaranteeing the provision of health-care services [7]. Although it was officially declared no longer a global threat [8], pandemic had left a deep change in the general adoption of hygiene rules, after having shown the fragility of public health services and the importance of implementation of adequate protective measures to contain the spread of the virus [9].

In the CL field, the debate about protective measures was notably intense [[10], [11], [12]]. Due to the close working distance between practitioners and patients, the potential for COVID-19 transmission posed a substantial challenge, profoundly affecting clinical tasks [[13], [14], [15]]. For instance, Italian CL practitioners reported an average reduction of new CL fittings and aftercare visits of 24.8 % and 17.9 % respectively compared to pre-pandemic number, while during the lockdown new CL fitting were temporarily suspended and aftercare visits were limited to very important eye conditions [16,17]. Furthermore, other than the inherent risks associated with close physical proximity, the concern about potential infections was amplified by the findings that SARS-CoV-2 had been isolated in tears and conjunctival swabs in patients affected by COVID-19 [[18], [19], [20]]. Nonetheless, the consistency in implementing protective protocols for both ECPs and CL wearers displayed significant variance, mirroring the variability observed in the adoption of compliant behaviours exhibited by CL wearers prior to the pandemic [16,21,22].

Numerous frameworks in psychological studies have been proposed to assess adherence to protective behaviours guidelines in health-related fields. The Health Belief Model (HBM) has been largely employed as a framework for understanding and guiding health-related interventions [23,24]. The model posits that an individual's decision to engage in a health-related behaviour (e.g., implementing protective measure) is influenced by the perception of personal susceptibility to develop a condition and the perceived severity of that condition, the perceived benefits and the perceived barriers in implementing a preventive behaviour, and the confidence in their ability to implement that behaviour [23,25].

The Integrated Belief Model (IBM) is a composite theoretical framework derived from the convergence of multiple social cognitive models (including HBM, Theory of Reasoned Action, and Theory of Planned Behaviour), indicating that the intention to perform a specific behaviour is influenced by the attitudes towards the behaviours, the perceived norms around the behaviour, and perceived personal control over the behaviour [24,26].

Those psychological/behavioural models have also been applied in CL field. Specifically, they have been employed to delve into the factors driving non-compliance in CL wear [27,28]. In addition, it has been found that employing more than one of these frameworks may help to identify diverse factors, potentially devising combined strategic interventions to reduce non-compliance rates in CL replacement by wearers [29]. Although health belief models (e.g., HBM) have been used to assess ECPs' risk perceptions regarding COVID-19 and identify components that might drive the adoption of preventive health behaviours in a general eye hospital environment [30], there have not been evaluations tailored specifically to understand CL practitioners' responses to the pandemic.

Hence, this study aimed to explore behaviour of ECPs in response to the COVID-19 pandemic, analysing the factors that influenced their intent to adhere to COVID-19 protective measures and their decisions to resume professional practice post-lockdown (i.e., performing new CL fitting and after care visit). Alongside providing insight into ECPs' responses in critical situations, HBM and IBM can also function as predictive tools for developing new protocols in routine practice and pinpointing essential factors to strengthen communication strategies.

## 2Methods

### 2.1 Questionnaire

In June 2020, following the end of Italy's first lockdown, an online survey targeting Italian ECPs was launched. At the time of the study many institutions had reduced capacity in their ethical committees. This study was affected by this, and the ethics committee deemed due to anonymity of survey, it was exempt from requiring full ethical committee approval. Nonetheless the tenets of Helsinki were adhered to throughout the study. Assottica Gruppo Contattologia, an association uniting Italy's primary CL manufacturers and distributors in Italy, extended the invitation to ECPs through Qualtrics (Qualtrics XM, Seattle, USA). ECPs with a minimum of one year's experience in CL practice were invited to participate. No financial incentive was offered, and participation was entirely voluntary and anonymous, with informed consent being obtained before the start of the questionnaire. To ensure the survey's effectiveness and clarity, a questionnaire was produced and refined by a focus group consisting of seven ECPs, four with less than three years of clinical experience and three experienced practitioners with over a decade of experience. The questionnaire was then further reviewed by this group plus two additional ECPs with more than a decade of clinical experience plus experience of writing and evaluating questionnaires. The questionnaire was structured into five sections.

#### 2.1.1 Demographic and professional data

This segment comprised seven questions about participants' age, gender, location of practice, professional role, practice type, years in CL practice.

#### 2.1.2 New CL fittings and aftercare visits: “immediately post lockdown behaviour” and “future intentions”

The “*immediately post lockdown behaviour*” in terms of new CL fittings and aftercare visits was measured with two separate items, asking how many times the participants had performed new CL fittings and aftercare visits since the beginning of the phase 2 of the lockdown (immediately after the full lockdown). The participants responded on a 7-level Likert scale: (never, 1–2 times, 3–4 times, 5–6 times, 7–8 times, 9–10 times, and more than 10 times).

*“Intention to perform new CL fittings”* and *“Intention to perform aftercare visits”* after lockdown were measured by asking participants how much they were going to conduct new CL fittings and aftercare visits in the next two weeks, regardless of the patients’ demand. The participants responded on a 7-level Likert scale ranging from 1 “Not at all” to 7 “Totally”.

#### 2.1.3 Protective measures during new CL applications and aftercare: “*immediately post lockdown behaviour*” and *“future intentions”*

As far as concern the protective measures, the “*immediately post lockdown behaviour*” and the *“future intention”* were explored. A description of the specific protective measures (use of personal protective equipment, adopt adequate behaviour in terms of distance to take during practice, and how to set environment and interaction in the office spaces) was provided based on the guidelines from a scientific committee of the voluntary, inter-associative register for optometrists and opticians, gathering the main Italian associations in the field [31].

*“Immediately post lockdown behaviour*” was examined by two different items asking participants how frequently they had implemented the protective measures during new CL fittings and aftercare visits since the beginning of the phase 2 of the lockdown (immediately after the full lockdown). The participants responded on a 7-level Likert scale ranging from 1 “Never” to 7 “Always”. An option to indicate that no new fitting/aftercare visit were performed was available.

Intention to comply with protective measures during new CL applications and aftercare visits was explored with two different items which asked them to indicate how likely they were going to apply protective measures during new CL fittings and aftercare visits in the next two weeks. The participants responded on a 7-level Likert scale ranging from 1 “Not at all likely” to 7 “Quite likely”. An option to indicate that no protective measures were going to be implemented was available.

#### 2.1.4 Health belief model (HBM)

This section included 15 items explored the four dimensions of the classic HBM theory (Appendix A): perceived threat (three items for susceptibility and three items for severity), perceived benefits (three items), perceived barriers (four items), and perceived self-efficacy (two items). The participants responded indicating their agreement with different statements on a 5-point Likert scale ranging from 1 “Strongly disagree” to 5 “Strongly agree”.

#### 2.1.5 Integrated Behavioural Model (IBM)

This section included 28 items, 14 referring to new CL fittings and 14 to aftercare visits. They explored the three dimensions of the IBM theory: attitudes (four items), perceived norms (six items), and personal agency (four items). The participants responded with their level of agreement with different statements on a 7-point Likert scale with a statement on a scale ranging from −3 “Strongly disagree” to +3 “Strongly agree” for the perceived norms and personal agency dimensions. For the attitudes dimension, instead, the participants responded on semantic differentials ranging from −3 (negative meaning) to +3 (positive meaning). All the items and the relative scale responses are reported in Appendix B.

**Fig. 1:**
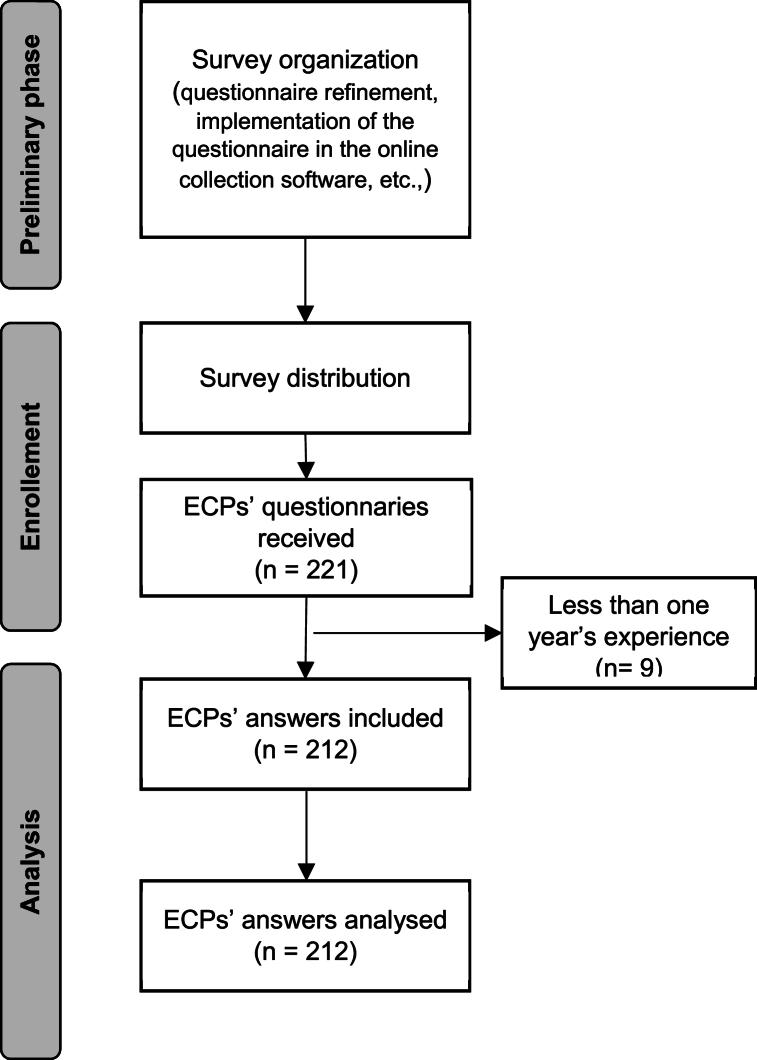
STROBE Flow diagram.

The flow chart of the study, which follows the Strengthening the Reporting of Observational Studies in Epidemiology (STROBE) statement, is shown in Fig. 1.

### 2.2 Data analysis

To predict the behavioural intention to undertake new CL fittings or aftercare visits following lockdown and to comply with COVID-19 protective measures three-model hierarchical regression analysis (HRA) were performed entering progressively into the analysis different group of variables. In the first model, age, sex, and years of CL practice (from hereafter “background variables”) were entered to control for effects on the outcome variables. In the second model, a further variable (the *immediately post lockdown behaviour*) was integrated as a reliable predictor of individual behaviour [32]. Finally, in the third model, the IBM and HBM variables were included in the regression equation. More specifically, IBM variables were entered to predict the behavioural intention to undertake new CL fittings or aftercare visits following lockdown, whereas HBM variables were entered to predict the compliance with COVID-19 protective measures.

To interpret the results, the F-test statistic (*F*) was used to evaluate the overall significance of the models. Probability values (*p-value*) below .05 were considered acceptable for statistical significance. The explanatory power of the models was measured by the coefficient of determination (*R*^*2*^), while increases in explained variance from the addition of new predictors were assessed using the change in *R*^*2*^ (*Delta R*^*2*^). Multicollinearity among independent variables was evaluated utilizing tolerance and variance inflation factor (*VIF*) values. Data were analysed using SPSS Statistics 25 (IBM, 2017).

## 3 Results

A total of 212 ECPs (150 males; 70.8 %) who completed the questionnaire were eligible for the study (Fig. 1). The mean age of the sample was 48.9 ± 12.0 years (range 22–76), with an average clinical experience in years of 23.8 ± 12.4 years (range 1–50). The participants demographics and practice information are reported in Table 1.

**Table 1:** Participant demographics and information about clinical practice (N = 212).

Age (yrs), Mean ± SD; (range)	48.9 ± 12.0; (22–76)
Gender, n (%)	Male 150 (70.8 %) Female 61 (28.8 %) No response 1 (.4 %)
Geographical area of clinical practice, n (%)	North 107 (50.5 %) Centre 55 (25.9 %) South 35 (16.5 %) Islands 14 (6.6 %) No response 1 (.5 %)
Professional title, n (%)	Dispensing Optician 63 (29.7 %) Optometrist with bachelor's degree 36 (17.0 %) Optometrist with diploma 94 (44.3 %) Optometrist with diploma and bachelor's degree 13 (6.1 %) Other 6 (2.8 %)
Prevalent type of practice n (%)	Independent in a high street practice 174 Higher education 4 Local retail chain 23 Regional or national retail chain 11
Years of CL practice, Mean ± SD; (range)	23.8 ± 12.4; (1–50)

### 3.1 New CL fittings and aftercare visits: “immediately post lockdown behaviour” and “future intentions”

**Fig. 2:**
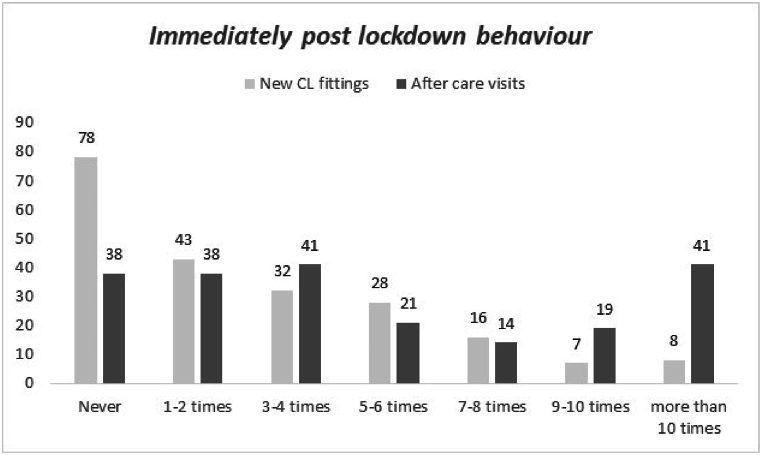
Distribution of new CL fittings and after care visits performed by interviewees immediately after the lockdown (N = 212).

The distribution of new CL fittings and after care visits performed by interviewees immediately after the lockdown is reported in Fig. 2. Mean ± SD (median) resulted 2.6 ± 1.7 (2), and 3.7 ± 2.2 (3) for new CL fittings and after care visits respectively. A statistical difference between the distribution was found (Wilcoxon test; p < 0.0001) also a significant difference in terms of frequency distribution was found (chi^2^; p < 0.0001).

**Fig. 3:**
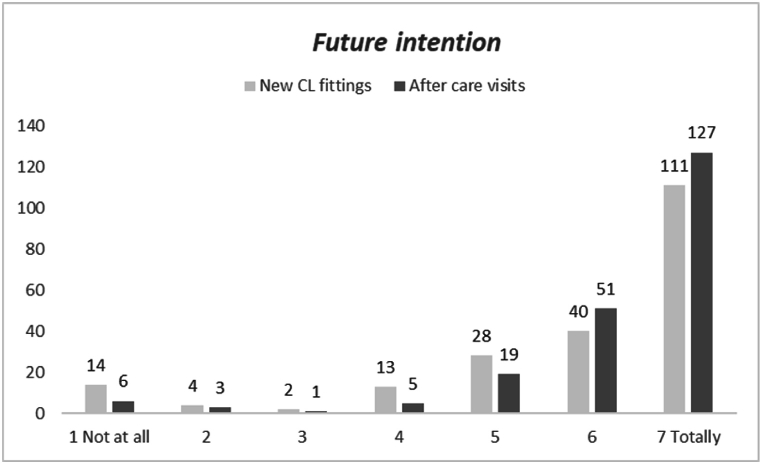
Distribution of intention score to perform new CL fittings and after care visits declared by interviewees (N = 212).

The distribution of scores of “*Intention to perform new CL fittings and aftercare visits”* is reported in Fig. 3. Mean ± SD (median) resulted 5.8 ± 1.7 (7) and 6.3 ± 1.3 (7) for new CL fittings and after care visits respectively. A statistical difference between the distribution was found (Wilcoxon test; p < 0.001). No significant difference in terms of frequency distribution was found (chi^2^; n.s.).

### 3.2 Protective measures during new CL applications and aftercare: “immediately post lockdown behaviour” and “future intentions”

**Fig. 4:**
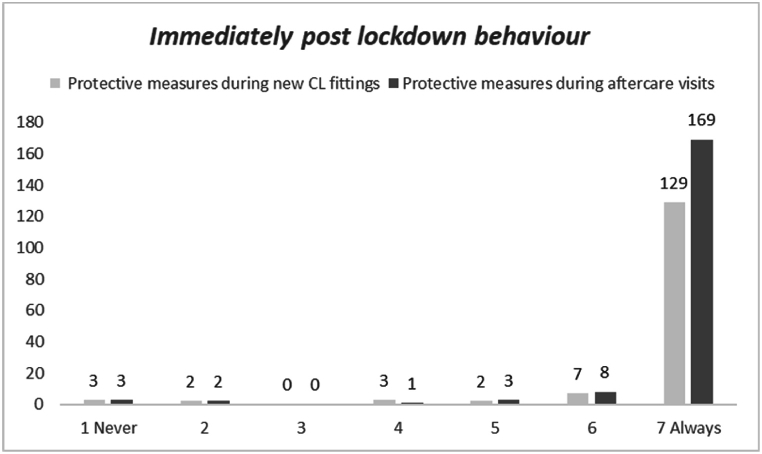
Distribution of the frequency of implementing protective measures in new CL fittings and after care visits performed by interviewees immediately after the lockdown (N = 212). Sixty-six and twenty-six interviewees declared that did not perform new fittings and aftercare visits respectively.

The distribution of the implementing of protective measures during new CL applications and aftercare performed by interviewees immediately post lockdown is reported in Fig. 4. Sixty-six and twenty-six interviewees declared that did not perform new fittings and aftercare visits, respectively. Mean ± SD (median) resulted 6.7 ± 1.1 (7) and 6.8 ± 1.0 (7) for new CL fittings (N = 146) and after care visits (N = 186) respectively. A statistical difference between the distribution (N = 143) was found (Wilcoxon test; p = 0.05). No significant difference in terms of frequency distribution was found (chi^2^; n.s.).

**Fig. 5:**
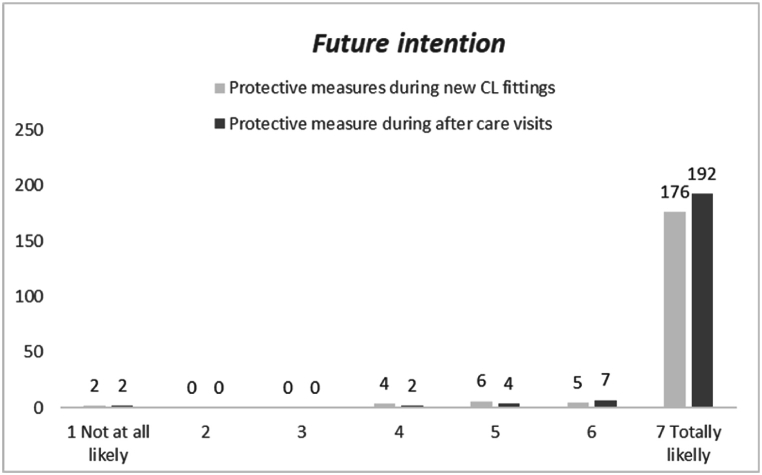
Distribution of intention scores to perform protective measures in new CL fittings and after care visits (N = 212). Nineteen and five interviewees declared that were not going to perform new fittings and aftercare visits respectively.

The distribution of the intention to implement protective measures during new CL applications and aftercare performed is reported in Fig. 5. Mean ± SD (median) resulted 6.8 ± .8 (7) and 6.8 ± .7 (7) for new CL fittings and aftercare visits respectively. No significant difference between the distribution (N = 193) was found (Wilcoxon test; n.s) as well as difference in terms of frequency distribution (chi^2^; n.s.).

### 3.3 Integrated Behavioural Model and intention to provide new CL fittings after lockdown

In Table 2 are reported the outcomes of the three-model HRA to evaluate the behavioural intention to provide new CL fittings after lockdown which incorporated in turn the background variables (age, gender, years of CL practice), the immediately post lockdown behaviour, and the IBM variables (attitudes, perceived norms, and personal agency). The first model (p < 0.005) revealed that the background variables explained 7.3 % of the outcome variable's variance. The second model (p < 0.001) suggested that past behaviour had a significant and positive relationship with the intention to provide new CL fittings (*Beta* = .41, p < 0.001). At this stage, the net change in the cumulative *R*^*2*^ was 15.1 % (*F change* = 40.23, p < 0.001). The third model (p < 0.001) indicated that past behaviour (Beta = .17, p = 0.01) maintained a significant and positive relationship with the outcome variable, as did attitudes (*Beta* = .49, p < 0.001). In contrast, personal agency, although marginally (*Beta* = .11, p = 0.05), was not significant related to the outcome variable, as they were not perceived norms (*Beta* = .06, *p* = 0.29). At this stage, the value of *R*^*2*^ increased to 49.3 % (*F change* = 36.12, p < 0.001). The tolerance value ranged between .21 and .92, and the VIF ranged between 1.09 and 4.69.

**Table 2:** HRA for intention to provide new CL fittings after lockdown (IBM).

Predictor	Beta	p-Value	Tolerance	VIF
**Model 1** (*R*^*2*^ = .07; *ΔR*^*2*^ = .07; *F*_*(3, 208)*_ = 5.45)				
Age	.05	.72	.21	4.65
Gender	.08	.29	.86	1.16
Years of CL practice	.19	.20	.21	4.64
**Model 2** (*R*^*2*^ = .22; *ΔR*^*2*^ = .15; *F(*_*1, 207)*_ = 40.23)				
Age	.07	.59	.21	4.65
Gender	−.01	.88	.82	1.21
Years of CL practice	.11	.39	.21	4.68
Past behaviour	.41	.00	.92	1.09
**Model 3** (*R*^*2*^ = .49; *ΔR*^*2*^ = .27; *F*_*(3, 204)*_ = 36.12)				
Age	.10	.34	.21	4.68
Gender	−.04	.47	.81	1.23
Years of CL practice	.09	.39	.21	4.69
Past behaviour	.17	.01	.75	1.34
Attitudes	.49	.00	.66	1.52
Perceived norms	.06	.29	.72	1.39
Personal agency	.11	.05	.80	1.25

**Notes**. R^2^ = Coefficient of Determination; ΔR^2^ = Change in R^2^; F = F-test Statistic; p-Value = Probability Value; VIF = Variance Inflation Factor.

### 3.4 Integrated Behavioural Model and intention to provide aftercare visits after lockdown

In Table 3 are reported the outcomes of the three-model HRA to evaluate the behavioural intention to provide aftercare visits following lockdown. The first model (p < 0.05) revealed that the background variables explained 4.8 % of the outcome variable's variance. Past behaviour, which was introduced in the second model (p < 0.001), contributed by 14.5 % variance to the cumulative *R*^*2*^ (*F change* = 37.25, p < 0.001). The second step indicated a positive and significant relationship between past behaviour and the intention to provide aftercare visits following lockdown (*Beta* = .39, p < 0.001). The third model (p < 0.001) indicated a positive significant relationship between attitudes and the outcome variable (*Beta* = .45, p < 0.001), with past behaviour remaining significant (Beta = .23, p < 0.001). However, none of the other IBM variables demonstrated a statistically significant relationship with the outcome variable. At this stage, the net change in the cumulative *R*^*2*^ was 20.6 % (*F change* = 23.37, p < 0.001). The tolerance value ranged between .21 and .93, and the VIF ranged between 1.08 and 4.79.

**Table 3:** HRA and intention to provide aftercare visits after lockdown (IBM).

Predictor	Beta	p-Value	Tolerance	VIF
**Model 1** (*R*^*2*^ = .05; *ΔR*^*2*^ = .05; *F*_*(3, 208)*_ = 3.52)				
Age	−.04	.80	.21	4.65
Gender	.06	.44	.86	1.16
Years of CL practice	.22	.12	.21	4.64
**Model 2** (*R*^*2*^ = .19; *ΔR*^*2*^ = .14; *F(*_*1, 207)*_ = 37.25)				
Age	.03	.84	.21	4.67
Gender	.01	.97	.84	1.19
Years of CL practice	.10	.97	.84	1.19
Past behaviour	.39	.00	.93	1.08
**Model 3** (*R*^*2*^ = .40; *ΔR*^*2*^ = .21; *F*_*(3, 204)*_ = 23.37)				
Age	.01	.90	.21	4.72
Gender	−.03	.60	.81	1.23
Years of CL practice	.11	.36	.21	4.79
Past behaviour	.23	.00	.79	1.26
Attitudes	.45	.00	.64	1.56
Perceived norms	.02	.73	.79	1.27
Personal agency	.06	.32	.72	1.38

**Notes**. R^2^ = Coefficient of Determination; ΔR^2^ = Change in R^2^; F = F-test Statistic; p-Value = Probability Value; VIF = Variance Inflation Factor.

### 3.5 Health belief model and intention to comply with COVID-19 personal protective measures during new CL fittings

In Table 4 are reported the outcomes of the three-model HRA to evaluate the behavioural intention to comply with COVID-19 personal protective measures during new CL fittings. Hierarchical regression analysis revealed a non-significant effect of background variables on the intention to comply with protective measures during new CL fittings (Model 1, *p* = 0.74). These variables accounted for .7 % of the variance in the intention to comply with protective measures (Table 4). When past behaviour was added to the regression equation (Model 2, *p* = 0.05), it contributed 5.6 % to the cumulative *R*^*2*^ variance (*F change* = 11.28, *p* = 0.001). The relationship between past behaviour and the intention to comply with protective measures during new CL fittings was positive and statistically significant (*Beta* = .25, *p* = 0.001). The third step (Model 3, p < 0.001) indicated that perceived benefits (*Beta* = .40, p < 0.001) and self-efficacy (*Beta* = .23, *p* = 0.001) had a significant and positive association with the intention to comply with protective measures during new CL fittings. There were no statistically significant associations between the outcome variable and perceived barriers (*Beta* = .07, *p* = 0.31) or perceived threat (*Beta* = .01, *p* = 0.91). In the third step, total variance increased to 28 % (*F change* = 16.57, *p* = 0.001). The tolerance value ranged between .22 and .92, and the VIF ranged between 1.08 and 4.51.

**Table 4:** HRA for intention to comply with COVID-19 personal protective measures during new CL fittings (HBM).

Predictor	Beta	p-Value	Tolerance	VIF
**Model 1** (*R*^*2*^ = .007; *ΔR*^*2*^ = .007; *F*_*(3, 189)*_ = .42)				
Age	.04	.78	.23	4.34
Gender	−.04	.61	.87	1.15
Years of CL practice	.05	.76	.23	4.41
**Model 2** (*R*^*2*^ = .06; *ΔR*^*2*^ = .06; *F(*_*1, 188)*_ = 11.28)				
Age	.06	.67	.23	4.35
Gender	−.08	.31	.85	1.18
Years of CL practice	−.01	.92	.22	4.47
Past behaviour	.25	.001	.92	1.08
**Model 3** (*R*^*2*^ = .31; *ΔR*^*2*^ = .25; *F*_*(4, 184)*_ = 16.57)				
Age	.06	.64	.23	4.41
Gender	−.01	.89	.82	1.22
Years of CL practice	−.04	.78	.22	1.22
Past behaviour	.17	.01	.82	1.22
Perceived benefits	.40	.00	.82	1.22
Perceived barriers	.07	.31	.87	1.15
Perceived threat	−.01	.91	.86	1.17
Self-efficacy	.23	.001	.78	1.28

**Notes**. R^2^ = Coefficient of Determination; ΔR^2^ = Change in R^2^; F = F-test Statistic; p-Value = Probability Value; VIF = Variance Inflation Factor.

### 3.6 Health belief model and intention to comply with COVID-19 personal protective measures during aftercare visits

In Table 5 are reported the outcomes of the three-model HRA to evaluate the behavioural intention to comply with COVID-19 personal protective measures during after care visits. Similarly to the previous HRAs, the step-1 model did not reveal a significant relationship between demographic variables and the intention to comply with COVID-19 personal protective measures during aftercare visits (Model 1, *p* = 0.93, Table 5). In the second step, past behaviour was added to the regression equation (Model 2, p < 0.001), and the value of *R*^*2*^ increased from 2 % to 18 % (*F change* = 42.80, p < 0.001). The relationship between past behaviour and intention to comply with protective measures during aftercare visits was positive and statistically significant (*Beta* = .43, p < 0.001). The step-3 model (p < 0.001) indicated that perceived benefits was the only HBM variable positively associated with the outcome variable (*Beta* = .31, p < 0.001). At this stage, the net change in the cumulative *R*^*2*^ was 10.6 % (*F change* = 7.32, p < 0.001). The tolerance value ranged between .22 and .95, and the VIF ranged between 1.05 and 4.61.

**Table 5:** HRA for intention to comply with COVID-19 personal protective measures during aftercare visits (HBM).

Predictor	Beta	p-Value	Tolerance	VIF
**Model 1** (*R*^*2*^ = .002; *ΔR*^*2*^ = .002; *F*_*(3, 203)*_ = .15)				
Age	.01	.93	.22	4.50
Gender	−.05	.50	.87	1.14
Years of CL practice	.01	.99	.22	4.52
**Model 2** (*R*^*2*^ = .18; *ΔR*^*2*^ = .17; *F(*_*1, 202)*_ = 42.80)				
Age	−.04	.74	.22	4.52
Gender	−.11	.12	.86	1.16
Years of CL practice	.00	.99	.22	4.52
Past behaviour	.43	.00	.95	1.05
**Model 3** (*R*^*2*^ = .28; *ΔR*^*2*^ = .11; *F*_*(4, 198)*_ = 7.32)				
Age	−.05	.72	.22	4.61
Gender	−.06	.34	.82	1.23
Years of CL practice	−.01	.93	.22	4.57
Past behaviour	.37	.00	.84	1.19
Perceived benefits	.31	.00	.77	1.30
Perceived barriers	.07	.29	.91	1.10
Perceived threat	.03	.61	.89	1.12
Self-efficacy	.04	.54	.73	1.37

**Notes**. R^2^ = Coefficient of Determination; ΔR^2^ = Change in R^2^; F = F-test Statistic; p-Value = Probability Value; VIF = Variance Inflation Factor.

## 4 Discussion

The main aims of this study were to investigate the impact of the COVID-19 pandemic on the clinical behaviour of Italian ECPs, analysing the factors that influenced the willingness and the modalities of restarting to perform new CL applications and aftercare visits after the lift of the 2020 lockdown restrictions, and their intent to adhere to COVID-19 protective measure. The responders were composed by a variety of professionals, with different educational background, and the ECPs were mainly located in optical stores, with independent practices representing the vast majority, suggesting an indicative depiction of CL services offered on a day-to-day practice in Italy [5]. Notably, none of the responders reported to be based in a hospital setting.

ECPs showed varying levels of commitment to conducting new CL fittings and aftercare visits. A general reduction in the number of new fittings was observed. Although the reducing frequency for higher number of new CL fittings reported by the professionals might coincide with a non-urgent context, the median frequency suggested a rate lower than that observed before the pandemic [5,16]. Notably, approximately 30 % of practitioners reported not performing any new CL applications. On the other hand, the frequency of aftercare visits displayed a more uniform distribution, although almost 20 % of ECPs not engaging in this aspect of their practice, suggesting that practitioners were more willing to continue providing care for existing wearers, rather than engaging with new fittings [16].

In terms of future intentions, the attitudes of the practitioners towards both new fittings and aftercare visits showed a similar pattern. The data revealed a skewness towards higher intentions for both activities, suggesting a readiness among professionals to resume more typical routines post-lockdown. Nevertheless, a minority of practitioners displayed a hesitation, more so for new fittings than for aftercare visits. Although it was not possible to determine whether this variance was driven more by the practitioners' choices or patient demand, it is plausible to infer that patient demand had a significant influence.

Meanwhile, ECPs demonstrated a strong commitment to employing protective measures during their services and showed an inclination to maintain these practices in the future.

Similar, instead, were the intentions of the practitioners in regard of performing new fittings and aftercare visits. For both the items explored, in fact, the scores collected from the ECPs were skewed towards the highest values, potentially indicating that the professionals were open to return to a more usual routine in practice after lockdown restriction lifting. Nonetheless, a small group of practitioners appeared reluctant, more towards new fitting than aftercare visits. Hence, despite it was not possible to ascertain if the difference was more determined by practitioners' decisions or wearers’ demand, it can be speculated that the latter was playing a more relevant role. Instead, ECPs appeared very dedicated in using protective measures in their practice, and willing to continue using them in the following period.

To investigate the more relevant factors underpinning practitioners’ behaviour and compliance towards implementing protective measures can help to understand the propensity of ECPs to implement recommended health behaviours in their clinical practice during emergency situations. Moreover, it can also indicate the key aspects related to the adoption of new rules, protocols, and, more generally, knowledge that will need to be implemented in everyday practice.

The IBM and the HBM have been used to investigate health beliefs of ECPs, aligning to procedure previously applied in the evaluation of hospital eye care service delivery [30], and other health behaviours in CL practice (i.e., compliance in daily disposable CLs replacement) [29]. The HBM was employed to predict the intention to comply with COVID-19 protective measures, given that compliance was considered a health-related behaviour [29]. The IBM was employed to assess the propensity that CL practitioners would maintain their professional practice following the lockdown. This is because a theoretical framework that analyses general behaviours is more effective at predicting non-health-related behaviours [33]. According with the Integrated Behavioural Model analysis, it could be noted that different factors could positively determine the intention to provide new CL fittings and aftercare visits after the lockdown. Past behaviour and positive attitudes toward the CL practice were the variable that predicted more strongly the intention to provide both new CL fittings and aftercare visits. ECPs expressing to perceive a higher self-accomplishment from their CL practice and viewing it as an essential service were more inclined to restore their usual practice, suggesting that more engaged practitioners are more willing to get back to their routine even in uncertain circumstances. Past behaviour was also found an important factor in influencing practitioners’ willing to provide CL services. Nonetheless, this factor seemed more crucial in resuming aftercare visits than in initiating new CL fittings. However, it is important to note that, although the provision of aftercare visits was significantly reduced during the lockdown, it was not completely interrupted, while, on the other hand, new CL fittings were almost completely halted during that period. Hence, the continuity in providing CL aftercare might explain the heightened emphasis on past behaviour when considering this service, as practitioners may have relied on the experience they had gained during the lockdown, in indicating their intention to restore CL aftercare visits. In light of this view, it can also be interpreted the reduced relevance of past behaviour reported by practitioners on restoring new CL fittings. ECPs may not have felt that they had gathered sufficient clinical experience during pandemic to rely on when expressing their opinion about performing new CL fittings after the lockdown.

To the same extent, it can be interpreted also the more influential role of personal agency, age and CL practice experience on the willing to perform new fittings, over aftercare visits. Given the paucity of directly acquired experience in the pandemic setting, the ECPs more inclined to perform new fittings may have relied on their confidence in abilities to perform CL fittings, and it can be speculated that this may be linked to higher experience, both personal and professional, although the factors were not statistically significant.

Similarly, the influence of personal agency, age, and CL practice experience on the inclination to conduct new fittings, compared to aftercare visits, can be understood. In the context of limited experience accumulated during the pandemic, ECPs more inclined to perform new fittings might have leaned on their confidence in their CL fitting abilities. It can be speculated that this confidence might be associated with more extensive personal and professional experience, even though these factors were not showing statistical significance.

The hierarchical regression analyses within the HBM for new CL fittings and aftercare visits reveal that past behaviour is the strongest predictor of future compliance for both types of appointment, emphasizing the role of habitual health practices. While demographic factors have minimal impact, the perceived benefits of protective measures significantly influence intentions to comply, more so than perceived barriers or threats. These results highlight the importance of effectively communicating these benefits, especially in emergency contexts, rather than focusing solely on potential dangers. Moreover, only in new CL fitting appointments, self-efficacy uniquely predicts compliance, suggesting that interventions should focus on empowering patient confidence by providing comprehensive information through visual aids and hands-on training. This instruction should be personalized to meet individual needs and concerns and ensuring ongoing support with follow-up care. Interestingly, perceived barriers and threats from COVID-19 are not significant predictors, indicating their limited role in influencing protective behaviours in CL care. The findings suggest that a tailored approach, leveraging past behaviours and perceived benefits, could enhance compliance strategies, with self-efficacy as a key focus for new patient interactions.

This study is subject to some limitations. First, none of the respondents were based in hospital settings. A more comprehensive understanding of ECPs' perceptions could be obtained by assessing these processes among professionals working not only in high street practices but also within hospital environments, where the perceived severity of the emergency was greater. Second, the cross-sectional design of the study precludes the possibility of inferring causality. A follow-up data collection, in which behavioural intention could serve as a predictor of actual behaviour, would offer valuable insights. The IBM model, for this purpose, encompasses additional variables (e.g., *cues to action* [24]) that were not included in the current study but should be considered in future research. Additionally, other background variables, such as socioeconomic status and cultural differences, could enrich the model's explanatory power. Finally, a deeper comprehension of the present findings might be achieved through a mixed-method study design, integrating both quantitative and qualitative approaches, such as interviews and focus groups.

Despite these limitations, the present study provides insights into the factors that may influence ECPs’ propensity to adopt protective measures during emergencies and to adapt their professional practices. These findings suggest that, even under uncertain conditions like those encountered in emergencies, there are actionable factors within the CL community that can support the efficacy of professional practice.

## CRediT authorship contribution statement

**Federica Scarci:** Writing – review & editing, Writing – original draft, Software, Formal analysis, Data curation. **Alfredo Desiato:** Writing – review & editing, Writing – original draft, Software, Formal analysis, Data curation. **Giulia Carlotta Rizzo:** Writing – review & editing, Writing – original draft, Software, Formal analysis, Data curation. **Stefano Livi:** Writing – review & editing, Writing – original draft, Supervision, Resources, Methodology, Conceptualization. **Mara Marini:** Writing – review & editing, Writing – original draft, Software, Formal analysis, Data curation. **Alessandra Cecalupo:** Writing – review & editing, Writing – original draft, Software, Formal analysis, Data curation. **Silvia Tavazzi:** Writing – review & editing, Writing – original draft, Supervision, Resources, Project administration, Methodology, Conceptualization. **Shehzad A. Naroo:** Writing – review & editing, Writing – original draft, Methodology, Conceptualization. **Fabrizio Zeri:** Writing – review & editing, Writing – original draft, Visualization, Validation, Supervision, Resources, Methodology, Formal analysis, Data curation, Conceptualization.

## Data availability statement

Data will be made available on request by contacting the corresponding author.

**APPENDIX A:** Items exploring Health belief model in the questionnaire. The participants responded with their level of agreement with a statement on a scale ranging from −3 “Strongly disagree” to +3 “Strongly agree”

HBM dimension	Statement
**Perceived threat: S*usceptibility***	The possibility of me developing a COVID -19 infection is very high.
	I feel that there is a good chance that I will develop a COVID -19 infection in the future.
	My physical health condition makes me more susceptible to developing a COVID-19 infection.
**Perceived threat:** S***everity***	If I developed a COVID-19 infection, my whole life would suffer.
	If I developed a COVID-19 infection, I know I would have to face the consequences for a long time.
	If I developed a COVID-19 infection, my health would be seriously damaged.
**Perceived benefits**	Implementing protective measures allows preventing a possible/potential COVID-19 infection.
	Implementing protective measures is beneficial for my health and for that of the people around me.
	Implementing protective measures allows continuing practicing your profession by reducing risks.
**Perceived barriers**	Getting the tools/devices necessary to comply with the protection standards is expensive.
	Implementing protective measures involves an excessive waste of time.
	In this period, it is difficult to find the tools/devices necessary to comply with the protection standards.
	Developing the habit of implementing workplace safety regulations/guidelines is difficult.
**Perceived self-efficacy**	I feel able to apply the protection rules when carrying out contact lens fitting to new wearers.
	I feel able to apply the protection rules when carrying out contact lens aftercare on existing wearers.

**APPENDIX B:** Integrated Behavioural Model. The participants responded with their level of agreement with a statement on a scale ranging from −3 “Strongly disagree” to +3 “Strongly agree” for the perceived norms and personal agency dimensions. For the attitudes dimension the participants responded on semantic differentials ranging from −3 (negative meaning) to +3 (positive meaning).

Dimension	Statement
**Attitudes**	For me, performing *new fitting/contact lens aftercare* would be: Unpleasant/Pleasant
	For me, performing *new fitting/contact lens aftercare* would be: Unsatisfactory/Satisfactory
	For me, performing *new fitting/contact lens aftercare* would be: Harmful/Beneficial
	For me, performing *new fitting/contact lens aftercare* would be: Useless/Useful
**Perceived norms**	Colleagues from my country disapprove performing *new fitting/contact lens aftercare*
	Colleagues from abroad disapprove performing *new fitting/contact lens aftercare*
	People close to me (relatives and friends) disapprove performing *new fitting/contact lens aftercare*
	Medical experts disapprove performing *new fitting/contact lens aftercare*
	Colleagues from my country continue performing *new fitting/contact lens aftercare*
	Colleagues from abroad continue performing *new fitting/contact lens aftercare*
**Personal agency:** ***Control***	Regardless of COVID-19, I think I have control over the performing of *new fitting/contact lens aftercare*
	Regardless of COVID-19, maintaining control over the performing of *new fitting/contact lens aftercare* is difficult
**Personal agency:** ***Self-efficacy***	Regardless of COVID-19, I feel capable of performing *new fitting/contact lens aftercare*
	Despite the difficulties associated with COVID-19, I know I can perform *new fitting/contact lens aftercare*

## Funding sources disclosure

This research did not receive any specific grant from funding agencies in the public, commercial, or not-for-profit sectors.

## Declaration of competing interest

The authors declare that they have no known competing financial interests or personal relationships that could have appeared to influence the work reported in this paper.
